# Prognostic impact of diagnostic and therapeutic time delays in breast cancer: an exploratory data analysis for patients at Parirenyatwa Hospital, Zimbabwe

**DOI:** 10.4314/ahs.v24i3.20

**Published:** 2024-09

**Authors:** Bester Saruchera, Oliver Bodhlyera, Henry Mwambi, Ntokozo Ndlovu

**Affiliations:** 1 School of Mathematics, Statistics and Computer Science, University of KwaZulu-Natal, Private Bag X01, Scottsville 3209, South Africa; 2 Department of Oncology, Faculty of Medicine and Health Sciences, University of Zimbabwe, P.O.Box MP167. Mt Pleasant, Harare

**Keywords:** Breast cancer, primary delay, secondary delay, treatment delay, Zimbabwe

## Abstract

**Objective:**

This paper seeks to investigate factors related to time delays for diagnosis and treatment in breast cancer patients at Parirenyatwa Hospital in Zimbabwe and subsequently evaluate the effects of presentation and diagnosis delays on cancer stage.

**Methods:**

The study was done for 379 patients with histologically diagnosed invasive breast cancer, from 2015 through to 2019. The study sought to identify factors associated with the time delays ( months)using parametric and non-parametric methods, depending on whether underlying assumptions of such tests are met. A multiple logistic regression model was also used to analyse the association between factors, primary delay, secondary delay variables and cancer stage.

**Results:**

The median of the primary, secondary and treatment delay were found to be 7.6, 1 and 0.4 months respectively. Rural residence, Karnofsky Performance Score below 70%, hypertension comorbidity, tumor size (>5cm) and well differentiated tumors (grade 1) were significant factors for delayed presentation. Longer primary delay times and post-menopausal status were associated with secondary delay. Advanced cancer stage at diagnosis and those on medical aid were more likely to have a delay in treatment onset.

**Conclusion:**

Primary and secondary delay were predictive of advanced disease using logistic regression.

## Background

Breast cancer is the most common female cancer across the world^1^. At the end of the year 2020, there were 7.8 million women alive who were diagnosed with breast cancer in the past 5 years. According to the World Health Organisation Breast Cancer Fact Sheet (2021), in 2020 alone, 2.3 million women were diagnosed with breast cancer globally and 685 000 deaths were reported^2^.

In a publication report by Chokunonga et al.^3^, 13.5% of new cancer cases were breast cancer while cervical cancer accounted for 37.1% among Zimbabwean black women. The most recent International Agency for Research on Cancer (IARC) data shows that mortality from breast cancer is highest in sub-Saharan Africa, with a five-year survival rate of less than 40% compared to a survival rate of 86% in thenited States^4^. Joko-Fru et al. 5 showed that rapidly increasing incidence rates of breast cancer over the past 15 years have been reported in Zimbabwe. Lack of breast cancer screening programs, time delay in establishing the diagnosis and ultimately delays in starting appropriate treatment for patients are a cause for concern as they contribute to high mortality levels in Africa.

Prolonged waiting periods before breast cancer diagnosis and the onset of treatment are of clinical significance as these delays lead to disease progression, clinically poor performance status or treatment failure. Rivera-Franco and Leon-Rodriguez^6^ in their study on delays in breast cancer detection and treatment in developing countries, reported that poor survival rates were a result of high proportion of women presenting with late-stage disease. Furthermore, their study also demonstrated that the low survival rates were linked to lack of adequate diagnosis and treatment facilities.

Previous studies by Ren and Hassen^7^, ^8^ revealed that globally, research into breast cancer aetiology has expanded knowledge of the disease. However, promoting early detection is still the major focus in fighting breast cancer and reducing mortality. Whilst studies have been done to investigate delay factors for patients with breast cancer, the established factors have been diverse. Ren et al.^7^ conducted a breast cancer total time delay study in China and linked delay factors to marital status, smoking status, low-income level and self-health care. In a cross sectional study by^8^, it was concluded that delay in seeking treatment was high in Ethiopia. Jassem et al.^9^ established a strong association between total delay time and indicators of cancer advancement at diagnosis (tumor size, nodal spread and distant metastases). Most studies have identified different factors for delays in patient presentation to medical facilities and treatment initiation^10^, ^11^.

Three types of delays were considered in this study. These were primary and secondary delays defined as when an event took more than 3 months to occur and treatment delay when a patient took more than 1 month to initiate treatment. Primary delay was defined as a patient's delay in seeking medical attention after potential breast cancer symptoms have emerged. Secondary delay was when health system factors together with patient knowledge and attitude towards the recommended tests, resulted in time delay between initial presentation to a health centre and the time of confirmed histological diagnosis. Treatment delay was defined as treatment initiation delay from the time of diagnosis to the point of treatment commencement. All of these delays could result in poorer prognosis for women with breast cancer.

This study investigated the demographic, clinical and treatment characteristics associated with primary, secondary and treatment delays of breast cancer in Zimbabwean women. The aim of detecting breast cancer early is to diagnose its severity and give early treatment to enhance chances of long-term survival. Prognosis is less severe with early detection than when detection of the disease is at more advanced stages.

## Methods

### Data Description

The data for this study was extracted from the Parirenyatwa Radiotherapy Centre records, for patients with histologically diagnosed invasive breast cancer, from 2015 through to 2019. Patients were excluded if they had a previous history of other types of cancer. This could result in poor prognosis in patients with second primary cancer due to adverse effects from prior treatment or prior cancer recurrence. A population based study by Wang et al.^12^ concluded that prior cancer history is an important exclusion criterion from clinical trials as patients with prior cancer had an inferior survival compared to those without. Records with missing pertinent information on the dates of key diagnostic and treatment events and patients who never received any therapy were also excluded from the time delay analysis. Finally, 379 records were included in this study.

The study was approved by the Medical Research Council of Zimbabwe and Joint Research and Ethics Committee (JREC) of Parirenyatwa Group of Hospitals and University of Zimbabwe. Patients' data was de-identified for utmost confidentiality.

### Predictor variables

Variables collected included patients' demographics, diagnosis and diagnostic processes and treatment as shown in Table 1.

**Table 1:** Variables and measurements

**Demographic Variables**	age at presentation, marital status (married, single), place of residence (rural or urban), Menopausal status (pre, peri, post), number of children, age at the first birth, employment Status (yes/no), medical Aid use, comorbidities presence (Diabetes Mellitus(DM), hypertension(HPT), HIV), family history of breast cancer(yes/no)
**Clinical Variables**	tumor grade (1,2,3), tumor size (≤2 cm, (2cm,5cm and >5cm), laterality, histological subtype (Luminal A, Luminal B, Triple Negative,HER2 NEU positive), Karnofsky Perfomance Score (KPS), disease stage, site of metastases
**Treatment Variables**	Initial Treatment (surgery, neoadjuvant or palliative chemotherapy, External Beam Radiotherapy (EBRT))

Demographic Variables age at presentation, marital status (married, single), place of residence (rural or urban), Menopausal status (pre, peri, post), number of children, age at the first birth, employment Status (yes/no), medical Aid use, comorbidities presence (Diabetes Mellitus(DM), hypertension(HPT), HIV), family history of breast cancer(yes/no)

Clinical Variables tumor grade (1,2,3), tumor size (≤2 cm, (2cm,5cm and >5cm), laterality, histological subtype (Luminal A, Luminal B, Triple Negative,HER2 NEU positive), Karnofsky Perfomance Score (KPS), disease stage, site of metastases

### Treatment Variables Initial Treatment

(surgery, neoadjuvant or palliative chemotherapy, External Beam Radiotherapy (EBRT))

In this study, tumor size was categorised into category 1 (≤2 cm), category 2 (2cm, 5cm and category 3 (>5cm) to capture meaningful distinctions. Category 2 encompasses tumor sizes greater than 2cm (excluding 2cm) and up to 5cm (including 5cm). Tumor, node and metastasis (TNM) status was used to classify disease stage (1,2,3,4). Reference was made to the 8th edition of the American Joint Committee on Cancer (AJCC) on breast cancer staging in determining some missing clinical information^13^,^14^. Stage at diagnosis was subsequently simplified into early stage (1-2: localized disease) and late Stage (3-4: locally advanced and metastatic disease). The marital status characteristic which had four groups for married, widowed, single and divorced was converted into 2 categories: married and single. The single category was made up of the divorced, widowed and never married. It is women's practice to claim the single status when either divorced or widowed. Other researchers in medical studies including Ren et al.^7^ and Joung^13^ have also implemented the categorisation into single and married for analysis. Primary and secondary delays were also examined as potential predictor variables for treatment delay and stage at diagnosis.

### Outcome variables

The main outcome variables included primary delay, secondary delay and treatment delay. Primary delay (also denoted as D1) was defined as the difference between date of breast symptoms onset and the patient's initial presentation to a medical facility minus 3 months. Secondary delay (D2) was defined as the difference between the date of first presentation and date of diagnosis minus 3 months. Treatment delay (D3) was the difference between the date of diagnosis and the date of onset of initial treatment minus 1 month. The treatment initiation date was taken as the date of first course of treatment by surgery, chemotherapy or radiation. Some women (seventy-three) did not have the date for treatment initiation thus an assumption was made that they never had treatment for breast cancer.

### Missing data

Missing data reduces statistical power, cause bias in estimation of parameters and reduces the representativeness of samples^14^. Patients' records with missing dates were removed and no attempt was made at imputing them. Due to missing data on some variables, an imputation process was carried out during pre-processing using Multivariate Imputation by Chained Equations (MICE). The MICE package from python software was used for handling missing data as the assumption that variables to be used in the imputation procedure for the missing data are Missing Completely at Random (MCAR) was satisfied. According to Mera-Gaona et al. 15, in a study to demonstrate the positive impact of multivariate imputation on datasets with missing values, MICE was found to reduce bias in the feature selection process.

We compared the basic statistics of the variables with actual and imputed values. The comparison of the statistics showed that the mean and standard deviation for both instances were almost equal as shown in Table 2.

**Table 2:** Basic statistics of MICE Data Imputation

Variable	Actual Dataset Variable Mean (SD)	Imputed Dataset Variable Mean (SD)
Age at first birth (years)	21.3 (3.8)	21.2 (3.2)
KPS (%)	82.3 (12)	82.3 (11.4)
Number of Children	4 (2)	4 (2)
Tumor Size (cm)	5.7 (3.8)	5.7 (3.3)
**V**	**A**	**I**
A	2	2
K	8	8
N	4	4
T	5	5

*KPS = Karnofsky Perfomance Score

### Statistical methods

The statistical analysis was carried out using python software in 3 steps: data pre-processing, data visualisations and analysis.

Box-Cox transformed secondary and treatment delay violated the assumptions of normality which are necessary for parametric tests. Non-parametric Mann-Whitney U and Kruskal Wallis tests were used to identify factors affecting these delays at the 5% significance level. The variables associated with primary delay were investigated using ANOVA and the independent samples t-test on the basis of them having met the normality assumptions. The Levene's test was used to assess the equality of variances for the variables in univariate analysis to ensure the homogeneity assumption was not violated. In cases where predictor variables were found to be statistically significant in the ANOVA test, the Tukey post-hoc tests were used to ascertain where the differences came from. Chi-square tests for independence were used to explore the association between categorical predictor variables (patient and clinical) and stage at diagnosis. A multiple logistic regression model was fitted to assess the effects of the independent variables on the binary stage outcome. A two-tailed p value was considered significant.

### The General Linear Time Delay Model

ANOVA is a statistical procedure that tests for the difference in means in three or more independent groups. The null hypothesis for the ANOVA is

for all (1)

The model is given by

(2)

where is the common effect of the whole experiment. represents the random error term. The errors are independent random variables. is the delay outcome variable, with +1 predictors. The linearity, homoscedasticity, normality and independence assumptions must be met for the general linear model to be fitted.

### Logistic Regression Model for Breast Cancer Stage

In other breast cancer studies, including one by Hussein et al.16, cancer stage was an important predictor of delay outcomes. We therefore sought to develop a logistic regression model to predict stage (early or late presentation) in breast cancer patients based on patients' and clinical variables. Patient and secondary delays were also considered as predictor variables in the analyses. The logistic model with k-predictor variables is given as

(3)

where is the odds of an outcome occurring against not occurring, with defining probability of the outcome occurring. The variables are the independent predictor variables, is the regression constant, are the logit change with a unit change in the predictor variable. The log likelihood function given by

(4)

Logistic regression uses Maximum Likelihood Estimation (MLE) to obtain the model coefficients relating the predictor variables to the outcome. The MLE is given by

(5)

## Results

**Figure 2:**
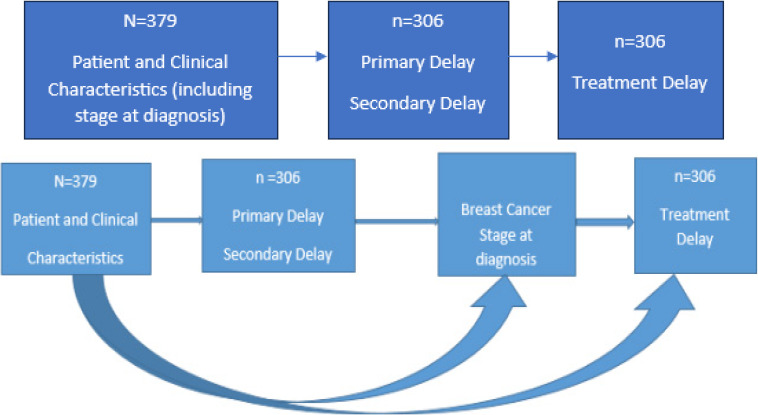
Flowchart highlighting schema of causations in time delay analyses for patients at Parirenyatwa Radiotherapy Centre, Zimbabwe (2015-2019)

Early diagnosis of breast cancer can be helpful in reducing adverse outcomes. In this section, variables associated with time delays and late-stage presentation in breast cancer patients in Zimbabwe are analysed. When the present study was initiated, 379 patients' records were used but 73 cases were then dropped in the analysis of delay due to missing treatment dates. Stage analysis was done for the 379 records whereas delay analysis was based on the 306 records with treatment initiation as shown in the flowchart in Figure 2.

Results of delay characteristics with patient and clinical variable characteristics are presented. This is followed by the presentation of the variable characteristics by stage at diagnosis. Generalised linear models including multiple logistic regression were used to analyse how stage at diagnosis are affected by primary and secondary delays.

### Delays characteristics

The next discussion highlights the patient and clinical characteristics by each delay type based on patients who only had treatment initiated.

Table 3 gives the basic descriptive statistics of the delay outcome variables. Primary delay interval time was the longest, whilst secondary and treatment delay exhibited similar means of approximately 3 months. Notably, even after having a diagnosis confirming the presence of breast cancer, patients at Parirenyatwa Radiotherapy Centre still delayed in initiating treatment (Table 3).

**Table 3:** Basic description of delay outcome variables

Delay Type	Mean	Median	Minimum	Maximum	Standard Deviation
Primary delay (months)	13	7.6	0.03	92.4	15.1
Secondary delay (months)	3.1	1	0.1	43.8	5.8
Treatment delay (months)	3	0.4	0.03	61.9	6.8

**Figure 3:**
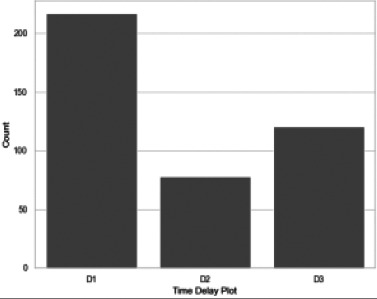
Delay Count Plots. D1, D2 and D3 represent the number of patients who experienced primary, secondary and treatment delay

Figure 3 gives the count plots for the time delays. The most frequent type of delay experienced by patients is the primary delay represented by D1.

### Primary delay time

Table 4 shows the results of the analysis of prognostic factors associated with delayed primary presentation using the independent sample t-test and ANOVA. The mean time for primary delay was found to be 13 months with the median time at 7.6months (Table 3). Primary delay was experienced by 71.2% of the patients. Patients with well differentiated tumors (grade 1) had the longest primary mean delay of 20.3 months compared to 11.3 months for those with poorly differentiated tumors (grade 3). The primary mean delay for rural patients of 14.4 months was higher than that of uban counterparts. Longer primary delays were associated with KPS), large tumor size (, rural residence (and well differentiated tumors (Whilst hypertension had a p value slightly above the 5% threshold (p, it was also considered as a plausible predictor. Some other relevant primary characteristics related to the patients such as age, employment status and family history of breast cancer did not significantly impact on the primary delay interval.

**Table 4:** Primary delay based on patient and clinical characteristics

Variable	Mean (SD)	ANOVA F / T-TEST		value
**Age**				
≤39	9.2 (8)			
40-59	13.5 (14.9)	1.193	0.305	
60	12.8 (17.3)			
**Marital Status**				
Single	11.6 (13.8)	1.180	0.239	
Married	13.8 (15.6)			
**A**				
	9			
	1	1		
6	1			
**M**				
	1	1		
	1			
**Menopausal Status**				
Pre	15.9 (19)			
Peri	12.8 (16)	0.316	0.730	
Post	11.9 (12)			
**Age at First Birth**				
≤25	12.2 (14.1)	1.337	0.182	
>25	15.2 (17.6)			
**Residence**				
Rural	14.4 (16)	2.216	0.027	
Urban	11.1 (13.2			
**Number of Children**				
**<5**	12.1(13.4)	0.531	0.596	
≥**5**	14.1(17.8)			
**FHBC**				
No	13.4 (15.6)	1.074	0.284	
Yes	9.4 (9.4)			
**EMPLOYMENT STATUS**				
No	13 (15.1)	0.885	0.377	
Yes	11.2 (12.8			
**Medical Aid**				
No	13.5 (15.9)	0.991	0.323	
Yes	11.2 (10.7)			
**Multiple Comorbidities**				
DM, HIV, HPT	12.3 (12.4)			
DM, HPT	13 (17.8)		0.540	0.658
HIV, HPT	13.1 (14.9)			
None	12.6 (14.5)			
**DIABETES MELLITUS**				
No	12.6 (14.5)	0.271	0.787	
Yes	13.2 (17)			
**HIV**				
No	12.4 (14.7)	1.097	0.274	
Yes	14 (14.4)			
**HPT**				
No	13.4 (14.4)	1.657	0.090	
Yes	11.3 (15.1)			
**Tumor Size (cm)**				
≤2	12.4 (17.2)	2.888	0.057	
(2,5	11.6 (14.5)			
>5	13.8 (13.8)			
**T**	1			
<	1	2		
	1			
5				
**KPS (%)**				
70	16.5 (18)	2.712	0.007	
>70	11.7 (13.6)			
**Tumor Grade**				
1	20.3 (25.5)			
2	13.2 (13.3)	2.934	0.054	
3	11.3 (13.6)			
**Histological Subtype**				
Luminal A	11 (10.5)			
Luminal B	12.6 (15.3)			
Triple negative	9.7 (11.5)	1.351	0.251	
Her2 Positive	9.2 (9.5)			
Unknown	15.9 (16.7)			
**Laterality**				
Left	12.9 (19)	0.339	0.735	
Right	12.3 (13)			

### Secondary delay time

Factors with significant effect on secondary delay were investigated. The mean time for this delay interval was found to be 3.1 months with a median of 1 month and 26.1% of the patients experienced secondary delays. Different menopausal status lead to statistical significant differences with secondary time delay associations with the peri and post-menopausal having the highest mean delay of around 3.2 months and which is slightly above the significant threshold. Having a primary delay was also associated with a secondary delay in breast cancer patients (0.03) as shown in Table 5. Whilst there was no statistical significance between comorbidities and secondary delay, breast cancer patients with multiple comorbidities of diabetes mellitus and hypertension had high secondary mean delay of 4.6 months. The results also show that being on medical aid or not did not make a difference in secondary delay times.

**Table 5:** Secondary delay based on patient, clinical and primary delay characteristics

Variable	Mean (SD), months	Kruskal Wallis/Mann Whitney test p value
**Age (Years)**		
<≤39	2.1 (2.7)	
40-59	2.9 (4.9)	0.850
>60	3.9 (8)	
**Marital Status**		
Single	2.8 (4.8)	0.562
Married	3.3 (6.3)	
**Menopausal Status**		
Pre	1.2 (2.5)	
Peri	3.1 (6.1)	0.064
Post	3.2 (5.2)	
**Age at First Birth (Years)**		
≤25	2.9 (5.2)	0.350
>25	3.5 (7.5)	
**Residence**		
Rural	3.3 (5.8)	0.664
Urban	2.8 (5.4)	
**Number of Children**		
**<5**	3 (5.4)	0.358
≥**5**	3 (6.1)	
**FHBC**	3 (5.6)	0.558
No	3 (5.7)	
Yes		
**EMPLOYMENT STATUS**		
No	2.9 (5)	0.354
Yes	3.3 (7.6)	
**Medical Aid**		
No	3 (5.1)	0.603
Yes	3 (6.6)	
**Multiple Comorbidities**		
DM, HIV, HPT	1.3 (1.6)	
DM, HPT	4.6 (8.5)	0.751
HIV, HPT	2.3 (3)	
None	2.9 (5.4)	
**DIABETES MELLITUS**		
No	2.9 (5.3)	0.827
Yes	3.8 (7.6)	
**HIV**		
No	3 (5.8)	0.883
Yes	2.8 (4.2)	
**HPT**		
No	2.9 (4.9)	0.679
Yes	3.2 (6.6)	
**KPS (%)**		
70	2.8 (4.2)	0.910
>70	3 (5.9)	
**Tumor Size (cm)**		
<2	2.9 (5.7)	0.163
(2,5	2.7 (5.5)	
>5	3.4 (5.6)	
**Tumor Grade**		
1	2.1 (2.4)	
2	3.3 (6.2)	0.974
3	2.9 (5.3)	
**Histological Subtype**		
Luminal A	3.2 (5.7)	
Luminal B	2.6 (4.9)	
Triple negative	2.6 (5.9)	0.335
Her2 Positive	3.6 (5.2)	
Unknown	3.2 (5.9)	
**Laterality**		
Left	3 (5)	0.133
Right	3 (6.2)	
**Primary Delay**		
No	3.6 (5.7)	0.031
Yes	2.8 (5.5)	

### Treatment delay time

An investigation of the treatment time was also done to identify the variables associated with such a delay. A total of 306 patients out of 379 (80%) had begun treatment according to the patients' records. Of the patients who had treatment initiated after diagnosis, 39% experienced delay. The mean time between diagnosis and treatment initiation was 3 months (2 months' delay) and median 0.4 months. Results in Table 6 show that patients without medical aid experienced a lower mean delay (2.5 months) compared to those with medical aid (4.1 months). The value of 0.015 suggested a statistically significant difference in treatment delay between those patients with and without medical aid. Notably the average delay for late diagnosis (3.3 months) was significantly higher than for early presentation (0.8 months), showing a significant association with treatment delay The site of tumor was of relevance, with patients with liver metastases (and bone metastases (likely to experience longer treatment delays than those with tumors in other locations).

**Table 6:** Treatment delay based on patient, clinical, primary and secondary delay characteristics

Variable	Mean (SD)	Kruskal Wallis/Mann Whitney test p value
**Age**		
≤**39**	1.6 (2.9)	
**40-59**	3.2 (7.4)	0.514
**>60**	3.1 (6.3)	
**Marital Status**		
**Single**	2.4 (4.9)	0.177
**Married**	3.6 (8.1)	
**Menopausal Status**		
**Pre**	3.0 (6.7)	
**Peri**	1.7 (2.1)	0.958
**Post**	3.1 (6.9)	
**Age at First Birth**		
≤**25**	2.9 (6.8)	0.413
**>25**	3.0 (6.0)	
**Residence**		
**Rural**	3.6 (8.1)	0.469
**Urban**	2.4 (5.1)	
**Number of Children**		
**<5**	2.9 (6.9)	0.441
≥**5**	3.1 (6.2)	
**FHBC**		
**No**	2.7 (6.5)	0.662
**Yes**	3.9 (7.1)	
**EMPLOYMENT STATUS**		
**No**	2.6 (5.3)	0.962
**Yes**	4.4 (10.5)	
**Medical Aid**		
**No**	2.5 (6.5)	0.015
**Yes**	4.1 (6.9)	
**Multiple Comorbidities**		
**DM, HIV, HPT**	0.6 (0.8)	0.793
**DM, HPT**	3.6 (7.2)	
**HIV, HPT**	13.5 (22.5)	
**None**	2.6 (5.7)	
**DIABETES MELLITUS**		
**No**	2.9 (6.7)	0.774
**Yes**	3.0 (6.4)	
**HIV**		
**No**	2.5 (5.0)	0.195
**Yes**	5.4 (11.4)	
**HPT**		
**No**	2.6 (5.3)	0.722
**Yes**	3.5 (8.4)	
**Tumor Size (cm)**		
≤**2**	3.5 (8.8)	0.419
**(2,5]**	2.8 (7.2)	
**>5**	2.8 (5.1)	
**KPS (%)**		
≤**70**	3.9 (9.3)	0.773
**>70**	2.7 (5.9)	
**Stage**		
**Early**	0.8 (1.7)	0.02
**Late**	3.3 (7.1)	
**Tumor Grade**		
**1**	2.5 (5.5)	0.635
**2**	2.9 (6.1)	
**3**	3.0 (7.2)	
**Histological Subtype**		
**Luminal A**	3.2 (8.3)	
**Luminal B**	2.0 (1.4)	
**Triple negative**	2.3 (4.0)	0.737
**HER2 neu positive**	1.5 (2.5)	
**Unknown**	3.8 (7.7)	
**Laterality**		
**Left**	2.3 (4.6)	0.569
**Right**	3.6 (8.2)	
**Bone Metastases**		
**No**	2.3 (5.9)	0.02
**Yes**	5.7 (10.1)	
**Liver Metastases**		
**No**	2.6 (6.3)	0.03
**Yes**	4.6 (8.4)	
**Lung Parechyma**		
**No**	2.8 (6.9)	0.07
**Yes**	3.5 (5.7)	
**Pleural Effusion**		
**No**	2.7 (6.3)	0.418
**Yes**	7.0 (11.4)	
**Spine Metastases**		
**No**	3.0 (6.8)	0.954
**Yes**	2.6 (3.8)	
**Primary Delay**		
**No**	1.9 (4.3)	0.200
**Yes**	3.4 (7.5)	
**Secondary Delay**		
**No**	3.3 (7.2)	0.506
**Yes**	2.0 (5.0)	

The results displayed in Table 10 demonstrated that tumor size was an important predictor, by which an increase in the size of the tumor was 1.253 times more likely to result in late stage disease at presentation. Women who give birth at ages above 25 years were 1.170 more likely to present with advanced disease. Peri menopausal Breast cancer patients were 2.2 times more likely to be diagnosed with advanced disease in comparison with pre-menopausal patients. Women with KPS more than 70% were 26 percent less likely to have late stage disease (O.R 0.74; 95% C.I -0.439 – 0.155). Effects of primary and secondary delay resulted in an increased risk of having a more advanced disease stage (3-4) (O.R 1.005, 95% CI 0.000-0.010, O.R 1.0133, 95% CI 0.003-0.023) respectively.

**Table 10:** Multiple Logistic Regression Model Results for Stage at diagnosis of Women with Breast Cancer at Parirenyatwa Radiotherapy Center, Zimbabwe, 2015-2019 (N= 379)

Variable		Odds Ratio	S.E.	95% C.I	
Age at First Birth	0.1571	1.170	0.07	0.019, 0.295	0.026
Primary Delay	0.0053	1.005	0.003	0.000, 0.010	0.040
Secondary Delay	0.0132	1.0133	0.005	0.003, 0.023	0.011
KPS	-0.2968	0.7431	0.072	-0.439, -0.155	0.000
Menopausal Status	0.7909	2.2053	0.296	0.211, 1.371	0.008
Tumor Size	0.2259	1.253	0.08	0.07, 0.832	0.005

## Discussion

The purpose of this study was to determine factors significantly associated with primary, secondary and treatment delays of breast cancer in women at Parirenyatwa Radiotherapy Centre in Zimbabwe and subsequently determine the predictors for late stage presentation. Longer waiting periods prior to breast cancer diagnosis and treatment initiation have significant prognostic impact on cancer stage progression, clinical deterioration, and/or higher risk treatment toxicity and interruptions. This unfortunately results in poorer prognosis for women with breast cancer. Early detection and diagnosis is poor in sub-Sahara Africa^17^,^18^. Other studies including Ren et al.^7^ reported that mortality is strongly linked to delays in diagnosis and treatment initiation. This study gave insights into the cancer situation at a government health facility in Zimbabwe with respect to breast cancer.

The proportion of primary delays observed 218 (71.2%) was significantly higher than in most studies in Africa^8^, ^19^. In a study conducted by Bhatia et al.^10^ involving 214 Botswana patients with breast cancer, 25.7% experienced primary delays. They identified associations between primary delay and level of education, employment status and severity of symptoms. Results in our study showed that unemployment was not associated with long delays which conforms to findings in other studies such as^7^,^9^. Our study revealed that 88% patients of the patients' diagnosed with late-stage disease experienced primary delay. Furthermore, it was observed that a primary delay exceeding 90 days was significantly associated with the presence of advanced breast cancer at diagnosis in Zimbabwe. In a multinational analysis by Jassem et al.^9^ for 12 European countries, the average primary related time delay was found to be 4.7 weeks. This was significantly shorter than the mean primary delay of 13 months (52 weeks) in this study. This could be attributed to several patient, health system and community factors. Patient knowledge and attitudes of breast cancer play a very significant role in the time delay between noticing a breast lump and eventual presentation at a health centre. Furthermore, the primary residence of a patient in relation to distance to the nearest functioning healthcare centre might also influence this decision as this has financial considerations. Rural patients were slightly more likely to experience a delay than urban ones. In this study, rural residents made up less than half the total number of patients and yet over 62% of Zimbabwe live in rural areas. This leads to suspicions that some rural patients do not even report cancer cases, rather than the assertion that rural communities have less cases of breast cancer. Whilst our results revealed a significant association between tumor size and delayed presentation, it is worthwhile to consider that the size of the tumor is likely as a result of delayed presentation and diagnosis and not a cause of it.

The mean secondary delay of 3.1 months in our study was comparable to other studies like that by Jassem et al.^9^ who had a mean for 3.1 months for Bulgaria, 3.6 months for Hungary, 3.1 months for Russia amongst the countries they carried out delay analyses for. In this study, shorter delay times were associated with early stage at diagnosis. They also found that higher educational level, older age and family history of breast cancer being significantly associated with shorter secondary time delays. Whilst residence was not a significant factor in secondary delay in this study, Hassen et al.^8^ found a significant association with diagnostic delay. In their same study in Ethiopia, they reported that traditional community interventions influenced delays in presentation and diagnosis. Our study did not analyse the use of traditional herbs as most patients could have misrepresented themselves by denying having resorted to such methods before seeking medical help. It is believed that most patients did not want to reveal that they had been resorting to the use of herbs as shown by 95% of patients indicating that they never used such. There is a general proliferation of traditional herbs purported to be cancer treatments sold on the streets of Zimbabwe and a lot of traditional healers publicly claim that they have cancer curing herbs. It is a common belief, among most women in developing countries, to use traditional remedies and spiritism treatment options before seeking medical attention. Other studies revealed that the use of traditional herbs was associated with delayed presentation^8^. Primary delay also significantly impacted secondary delay.

Our study also identified factors associated with treatment delay in breast cancer patients, shedding light in critical aspects influencing the timeous therapeutic interventions. The observed association between stage and treatment delay is particularly noteworthy. The finding of significantly higher mean delays in advanced stage compared to early stage (Table 6) aligns with existing literature emphasizing the urgency of timely treatment initation, especially in more advanced cancer stages^20^,^21^,^22^. The prolonged delays in the late stage patients warrant further investigations into the reasons behind these delays, with considerations for improved screening programs and public awareness campaigns. Our results also showed that metastases was significantly associated with the length of treatment delay. The result was not surprising, it is consistent with what is known about cancer biology, tumor doubling time and natural history of malignancy. Tumors grow in size and spread to regional lymph nodes and ultimately metastasise to distant organs over time. Ren et al.^7^ in a study which reviewed 298 Chinese patients reported a strong association between a time delay of 3 months and cancer diagnosis.

Contrary to expectations^22^,^23^, the study revealed that patients with medical aid experienced longer treatment delays. This disparity could be rooted in the study site being a public referral hospital. It is highly likely that patients on medical aid initially sought private health care before eventually seeking care at Parirenyatwa Hospital. The pathway from private to public healthcare may introduce additional processes, contributing to the observed delays. Although we expected employment status to be associated with delays in initiating treatment, as has been found in other studies including Ren et al.^7^ in China, this could not be concluded from this study. Most of the patients who visited Parirenyatwa Radiotherapy centre were lost to follow up (LTFU). A study in China showed that 26.8% were 5-year LTFU, with 12% LTFU in the first year^24^. Our results showed that LTFU was much higher compared with other studies in Africa. In similar studies, Nigeria, Namibia and Uganda had 0.8%, 2.2%, 5.6% LTFU at 3 years respectively^25^. This is a red flag that signals a weak health delivery system as the status of cancer patients must be followed up by the hospital. The growing burden of non-communicable diseases (NCDs) is gradually overwhelming specialist clinics and could possibly result in inadequate follow up capacity. The long queues observed at oncology centres in Zimbabwe could be a major turn off, despite them being inaccessible to the majority of the rural population. High travelling costs are also a major hindrance. Given the projection that NCDs are set to overtake communicable, maternal, neonatal and nutritional diseases combined as the leading cause of mortality by 2030 according to a publication by Bigna and Noubiap^26^, the system to reduce the LTFU rate need to be strengthened. Patients may also opt to see ‘alternativetherapy’ with herbalist and spiritualists due to the preconceived idea that cancer cannot be treated medically. Cancer is the subject of wide spread and varied mythology around the world, Africa included^27^.

Our results also showed that metastases was significantly associated with the length of treatment delay. The result was not surprising, it is consistent with what is known about cancer biology, tumor doubling time and natural history of malignancy. Tumors grow in size and spread to regional lymph nodes and ultimately metastasise to distant organs over time. Ren et al.^7^ in a study which reviewed 298 Chinese patients reported a strong association between a time delay of 3 months and cancer diagnosis. Primary delay also significantly impacted secondary delay.

The logistic model algorithm employed had a good accuracy level of 82% for the main predictors of stage at diagnosis which could have been much higher if records with missing dates were few, avoiding deletion of cases. A framework to deal with missing dates in clinical oncology data needs to be established in order to ensure that accurate models can be established.

## Conclusion

Time delays in presentation to a medical facility, diagnosis and treatment must be reduced. Research results indicated that rural residence, Karnorfsky Performance Score below 70%, hypertension comorbidity, tumor size(>5cm) and well differentiated tumors (grade 1) are significant factors for delayed presentation for patients attending Parirenyatwa Hospital in Zimbabwe. It was also observed that longer primary delay times were associated with secondary delays, a result that mean special attention should be rendered to those who delay to present themselves as they have high chances of delaying the next stages of treatment. From these results, we conclude that additional awareness campaigns targeting hypertension patients and rural woman will go a long way in curbing delayed presentations and subsequent delays thereafter. Continued breast cancer awareness programs must be put in place to enhance early detection and treatment initiation. Increasing radiotherapy centres in the country might also lessen the problems associated with patients not presenting themselves to cancer facilities. Our findings highlight the need for future work on impact of time delay on the survival of patients with breast cancer.

## Figures and Tables

**Figure 1:**
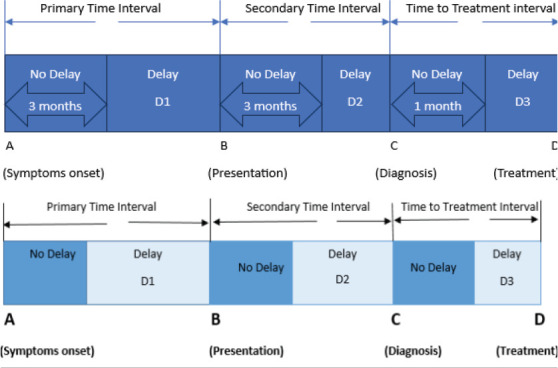
highlights the intervals showing delay periods from time of symptoms onset, presentation to a medical facility and initial treatment in breast cancer patients

**Table 7:** Chi-Square tests of the effects of Patient Characteristics of Women with Breast Cancer on Stage at diagnosis, Parirenyatwa Radiotherapy Centre, Zimbabwe in 2015-2019, (N=379)

Variable		Cancer Stage at diagnosis		Chi-square test	
	N(%)	Early (%)	Late (%)	χ^2^ (df)	p-value
**Age (years)**					
**>39**	68 (17.9)	8.8	91.2	1.887 (2)	0.389
**40-60**	216 (57)	14.8	85.2		
**>60**	95 (25.1)	15.8	84.2		
**Marital status**					
**Single**	175(46.2)	15.4	84.6	0.546 (1)	0.363
**Married**	204(53.8)	12.7	87.3		
**Menopausal status**					
**Pre**	158 (41.7)	9.5	90.5	5.020 (2)	0.081
**Peri**	28 (7.4)	21.4	78.6		
**Post**	193 (50.9)	16.6	83.4		
**Residence**					
**Rural**	173 (45.6)	13.9	86.1	0.003 (1)	0.954
**Urban**	206 (54.4)	14.1	85.9		
**Number of Children**					
**<=4**	231 (60.9)	13.4	86.6	0.546 (1)	0.459
**5+**	83 (21.9)	16.9	83.1		
**Age at First Birth**					
**<=25**	233 (61.5)	17.6	84.2	1.134 (1)	0.287
**25+**	28 (7.4)	3.6	96.4		
**FHBC**					
**No**	311 (82.1)	14.8	85.2	0.601 (1)	0.438
**Yes**	68 (17.9)	10.3	89.7		
**Employment Status**					
**No**	310 (81.8)	13.5	86.5	0.107 (1)	0.744
**Yes**	69 (18.2)	15.9	84.1		
**Medical Aid**					
**No**	284 (74.9)	14.1	85.9	0.009 (1)	0.922
**Yes**	95 (25.1)	13.4	86.3		
**Diabetes Mellitus**					
**No**	350 (92.3)	14.1	85.9	0.06 (1)	0.804
**Yes**	29 (7.7)	17.2	82.8		
**HPT**					
**No**	234 (61.7)	12	88	1.656 (1)	0.199
**Yes**	145 (38.3)	17.2	32.8		
**HIV STATUS**					
**NEGATIVE**	316 (83.4)	13.6	86.4	0.08 (1)	0.784
**POSITIVE**	63 (16.6)	15.9	84.1		
**Multiple Comorbidities**					
**HPT, HIV**	12 (3.2)	10	90	5.45 (3)	0.142
**HPT, DM**	22 (5.8)	18.2	81.8		
**DM, HIV, HPT**	3 (0.8)	33.3	66.7		
**Primary Time Interval**					
**No Delay**	105 (27.7)	19	81	2.541 (1)	0.1
**Delay**	274 (72.3)	12	88		
**Secondary Time Interval**					
**No Delay**	273 (72)	18.3	81.7	13.513 (1)	0.000
**Delay**	106 (28)	2.8	97.2		

**Table 8:** Chi-square tests of the effects of Clinical Characteristics of Women with Breast Cancer and stratification by Stage at diagnosis at Parirenyatwa Radiotherapy Centre, Zimbabwe in 2015-2019, (N=379)

Variable		Cancer Stage at diagnosis		Chi-square test	
	N(%)	Early (%)	Late (%)	χ^2^ (df)	p-value
**Tumor Size**					
**<3**	48 (16.3)	17	83	18.469 (2)	<0.001
**[3-5]**	131 (44.4)	17.2	82.8		
**>5**	116 (39.3)	9.2	90.8		
**Tumor grade**					
**1**	23 (6.1)	26.1	73.9	5.686 (2)	0.058
**2**	202 (53.3)	15.8	84.2		
**3**	154 (40.6)	9.7	90.3		
**KPS**					
**<=60**	31 (8.2)	0	100	9.039 (1)	0.002
**70+**	307 (81)	15	85		
**Histological subtype**					
**Luminal A**	75 (19.8)	9.3	90.7	11.716(4)	0.020
**Luminal B**	64 (16.9)	26.6	73.4		
**Triple Negative**	42 (11.1)	7.1	92.9		
**Her2 Positive**	25 (6.6)	16	84		
**Unknown**	173 (45.6)	12.7	81.3		
**Laterality**					
**Left**	197 (52)	13.7	86.3	0.197 (1)	0.906
**Right**	181 (47.8)	14.4	85.6		

**Table 9:** Treatment Characteristics of Women with Breast Cancer and LTFU at Parirenyatwa Radiotherapy Centre, Zimbabwe in 2015-2019, (N=379)

Variable	N(%)
**Initial treatment**	
**Masectomy**	177 (46.7)
**Neoadjuvant Chemotherapy**	62 (16.4)
**Palliative Chemotherapy**	47 (12.4)
**Palliative Radiotherapy**	17 (4.5)
**No Treatment**	75 (19.8)
**Radiation (EBRT)**	
**Curative Radiotherapy**	82 (21.6)
**Palliative Radiotherapy**	34 (9)
**Loss To Follow Up**	
**No**	25 (6.6)
**Yes**	354 (93.4)

## Data Availability

All the data required for the publication has been included in the research article. These are in the form of figures and tables in the research paper. The original dataset used and analysed in the study is available from the corresponding author upon request.

## Abbreviations

DM: Diabetes Mellitus
EBRT: External Beam Radiation Therapy
FHBC: Family history of breast cancer
HIV: Human immunodeficiency virus
HPT: Hypertension
HER2: Human Epidermal growth factor Receptor 2
IARC: International Agency for Research on Cancer
KPS: Karnofsky Performance score
LTFU: Loss to follow up
WHO: World Health Organisation
